# Electrospun Carbothane-Based Drug-Enriched Scaffolds for Cardiovascular Devices: Drug Release, Hemocompatibility, Endothelialization, and Immunological Characterization

**DOI:** 10.3390/ijms27115081

**Published:** 2026-06-04

**Authors:** Zhanna K. Nazarkina, Boris P. Chelobanov, Alena O. Stepanova, Aznaur Imenov, Pavel P. Laktionov

**Affiliations:** 1 Institute of Chemical Biology and Fundamental Medicine, Siberian Branch, Russian Academy of Sciences, Lavrentiev Ave. 8, Novosibirsk 630090, Russia; chelobanov.bp@mipt.ru (B.P.C.); lebedeva@1bio.ru (A.O.S.); a.imenov@g.nsu.ru (A.I.); lakt@1bio.ru (P.P.L.); 2 Moscow Center for Advanced Studies, Kulakova Str. 20, Moscow 123592, Russia; 3 V. Zelman Institute for Medicine and Psychology, Novosibirsk State University, Pirogov Str., Novosibirsk 630090, Russia

**Keywords:** sirolimus, electrospinning, drug release, vascular graft, drug-eluting stents, polyurethane, Carbothane, endothelialization, biocompatibility, hemocompatibility

## Abstract

Polyurethane (PU) is widely used in medical products due to its biocompatibility and mechanical properties. Electrospinning (ES) was employed to produce PU-based scaffolds intended for cardiovascular devices (CVD) from blends of Carbothane (Carb) with human serum albumin (HSA), dimethylacetamide (DMA), and drugs. Sirolimus (SRL)—an immunosuppressive/anti-proliferative drug—and diclofenac (DF)—a nonsteroidal anti-inflammatory drug—were introduced into ES blends to produce drug-enriched scaffolds that prevent inflammation and cell overgrowth. The biocompatibility, stability, and mechanical properties of the scaffolds and SRL release were studied. The scaffolds possessed good mechanical properties and were stable in PBS and blood plasma (BP) for 120 days. The minimal SRL release rate was observed for the scaffold 3%Carb/10%HSA/DMA/SRL. A study of scaffold interaction with blood demonstrated good hemocompatibility of most scaffolds. A study of human gingival fibroblasts, endothelial cells (HUVEC and EA.hy926), and vascular smooth muscle cell interaction with scaffolds in vitro demonstrated variability in cell viability and pro-inflammatory interleukin IL-6 secretion, depending on both the scaffold composition and the cell type. The incorporation of DF into scaffolds decreased the concentration of IL-6 in the culture medium. The scaffold 3%Carb/10%HSA/DMA/SRL is the best choice for CVD in terms of hemocompatibility, endothelialization, and the induction of minimal inflammation.

## 1. Introduction

A variety of synthetic polymers are used for the production of materials intended for cardiovascular surgery (CVS), such as expanded polytetrafluorethylene (ePTFE), polyurethane (PU), polyethylene terephthalate (PET), poly(D,L-lactide-coglycolide) (PLGA), polycaprolactone (PCL) co-polymers, and others [1,2]. Designing vascular grafts and stent coatings requires careful consideration of several parameters to replicate the mechanical and structural properties of native blood vessels. Materials intended for CVS must be non-toxic and exhibit good biocompatibility, biostability, and mechanical properties [3]. The biocompatibility includes the absence of toxicity, a lack of serious inflammatory reaction, the ability to promote endothelial cell adhesion and proliferation, a reduction in the platelet adhesion and aggregation, the absence of hemolysis, etc. Polyurethanes occupy the top positions in the list of polymers used in medical products for CVS due to their biocompatibility and mechanical properties, applicability in different manufacturing technologies, and the availability of different medical grade PU on the market. PU-based products include vascular access grafts VECTRA^®^ (Bard Inc., Murray Hill, NJ, USA), Silkothane^®^ (Dialybrid S.r.l., Cantù, Italy) [4,5], and the PK Papyrus coronary stent (Biotronik Inc., Lake Oswego, OR, USA) covered with Elast-Eon polyurethane [6]. However, such devices are not free from drawbacks. For example, vascular prostheses are usually offered mainly for arteriovenous anastomoses during hemodialysis rather than for peripheral bypass surgery [7]. Papyrus stents, despite their effectiveness and ease of use, are prone to stent thrombosis and restenosis [8,9,10]. There is an obvious need to improve their bio- and hemocompatibility. Options for improving these characteristics are discussed further in the text and are studied in the current work.

Polyurethane-based cardiovascular devices (CVDs) are intended for long-term implantation and must therefore satisfy several key requirements. Their long-term performance is primarily determined by mechanical properties, stability over time, and appropriate interactions with cells. Immediately after implantation, interaction with cells occurs against the background of an acute inflammatory reaction caused by surgery. Consequently, an ideal implant should not only avoid triggering a foreign body reaction but also suppress local or systemic inflammatory responses. In respect to CVS, inflammation causes neointima overgrowth and stenosis of implanted grafts or stents. Maintaining the stability of the polymer structure is obviously necessary for the long-term device functionality while maintaining its original properties.

The interaction of materials with cells depends on the chemical structure and on the meso- and nanoscale topography of the material surface. The chemical structure determines surface chemistry, hydrophilicity/hydrophobicity, and the ability to adsorb biomolecules. Both surface chemistry and surface topography affect cell adhesion, proliferation, and infiltration [11]. To enhance cell adhesion, natural polymers such as proteins are commonly used for surface modification. Electrospinning provides a unique opportunity to modify fibers—including their surface—with proteins by using blends of synthetic and natural polymers. Various drugs can be incorporated into the fibers via ES. PU-based scaffolds as well as scaffolds made from other polymers can be used for drug delivery [12] and could therefore also be applied for the production of drug-eluting stent coating. The introduction of antiproliferative and nonsteroidal anti-inflammatory drugs (NSAIDs) could reduce restenosis and attenuate the inflammatory response—both of which are major causes of postoperative complications in CVS. The release of the drugs from PU scaffolds depends on scaffold structure and stability, drug properties (solubility, hydrophobicity), and the drug-loading technique. Drug release from stable PU scaffolds occurs mainly via diffusion, and the release rate depends on the thickness, permeability, and drug solubility [13,14]. Drug release from the scaffolds can also be modulated by varying the composition of the ES solution [15,16]. ES appears to be a highly convenient method for producing biocompatible drug-enriched scaffolds due to generated fibers from multicomponent solutions or suspensions of synthetic polymers, natural polymers, and low-molecular-weight compounds.

The present work describes the fabrication and characterization of PU-based scaffolds intended for CVS. Carbothane™ AC-4075A (Carb), an aromatic, polycarbonate-based polyurethane (soft segment: polycarbonate; hard segment: aromatic isocyanate), was used. Carbothane™ AC-4075A is biostable, exhibits favorable mechanical properties, and is resistant to chemicals and solvents. Eight different scaffolds were fabricated via ES from blends of Carb with human serum albumin (HSA), dimethylacetamide (DMA), the antiproliferative drug sirolimus (SRL), and the nonsteroidal anti-inflammatory drug diclofenac (DF) in 1,1,1,3,3,3-hexafluoroisopropanol (HFIP). All scaffolds were evaluated for biocompatibility, mechanical stability, and SRL release.

## 2. Results

### 2.1. Fabrication and Characterization of the Scaffolds

**Figure 1:**
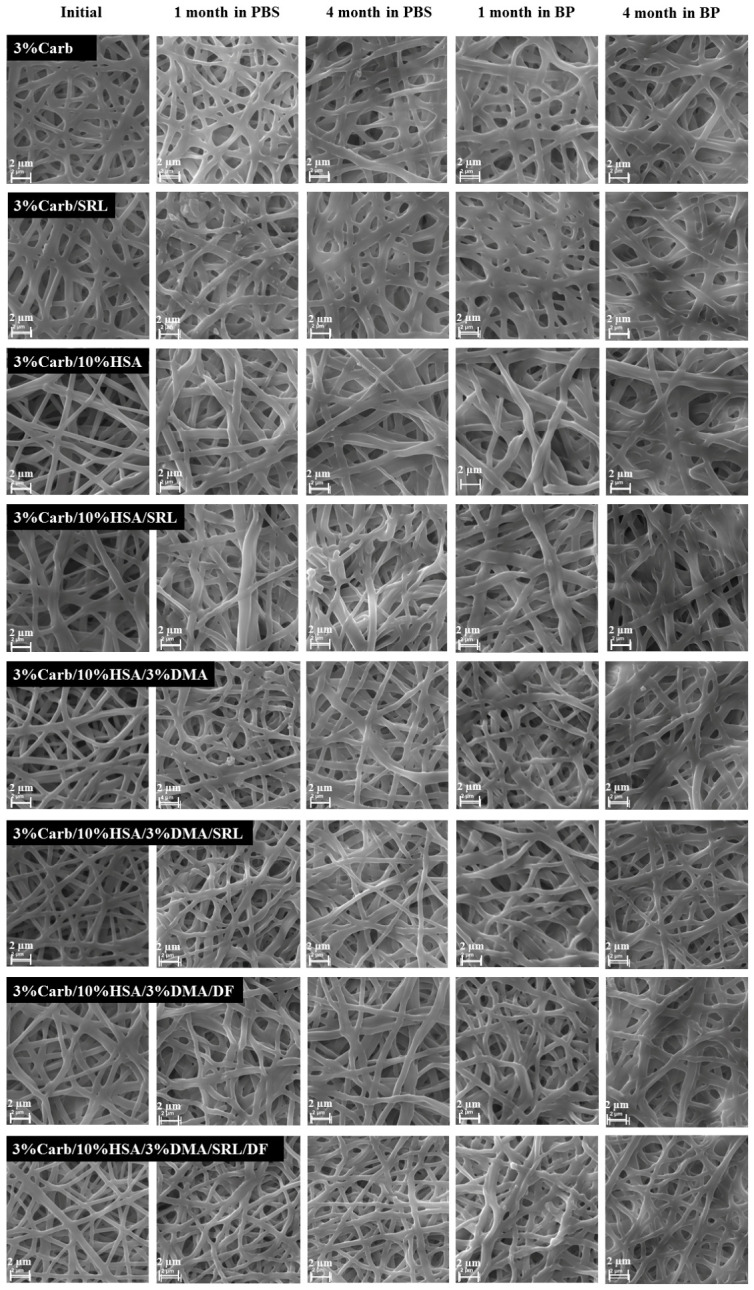
The influence of long-term incubation in PBS and BP on scaffold structure. SEM images of the Carb-based scaffolds before and after incubation of the scaffolds in PBS and BP at 37 °C for 30 and 120 days.

Polycarbonate urethane Carbothane AC-4075A was used as a main polymer to fabricate PU scaffolds without additives and composite scaffolds with HSA, DMA, SRL, and DF. HFIP was used as a main solvent for the preparation of ES blends. To decrease the solvent evaporation rate and affect the solubility of the compounds, DMA was added as an additional solvent. In total, eight different types of Carb-based scaffolds were fabricated using ES on a drum collector from solutions of PU with HSA, DMA, and drugs (please see the scaffold composition in Table 1). The structure of the scaffolds was studied by SEM (Figure 1). All scaffolds were composed of randomly oriented fibers. According to SEM at a higher resolution (×10,000), all scaffolds had a smooth fiber surface. The fiber diameter for Carb-based scaffolds varied from 508 ± 125 nm to 683 ± 166 nm (Table 1). The presence of the high-boiling polar solvent DMA in the ES solution resulted in a decrease in fiber diameter.

**Figure 2:**
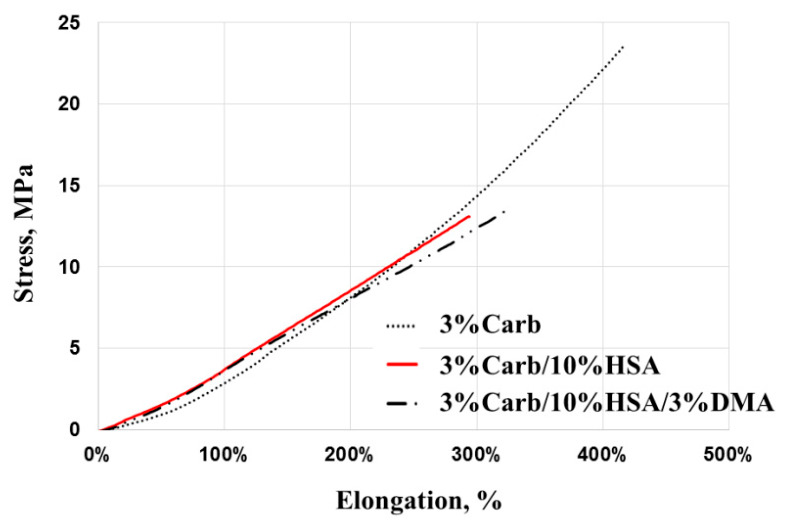
Typical stress–strain plots for scaffolds studied.

Preliminary experiments demonstrated that drugs do not affect the mechanical characteristics of the scaffold, so mechanical properties are presented for scaffolds without drugs (Table 1, Figure 2). The tensile strength of the scaffolds depends on the composition of the ES blends and varies from 15.1 ± 1.8 MPa to 24.6 ± 1.7 MPa. The elongation at break varied from 333 ± 44% to 427 ± 14%. The stress–strain curves for scaffolds 3%Carb, 3%Carb/10%HSA, and 3%Carb/10%HSA/DMA are very similar at the stretch range up to 250%. The scaffold 3%Carb is the most durable.

The stability of scaffolds structure was studied in vitro after incubation in PBS or in BP at 37 °C for 30 and 120 days, followed by scaffold structure SEM analysis (Figure 1). According to SEM data, incubation in PBS or BP for 30 and 120 days did not alter the structure of the scaffolds. There was no substantial scaffold degradation during the assayed time. We did not observe significant changes in fiber diameter after incubation (Table 1).

### 2.2. SRL Release

**Figure 3:**
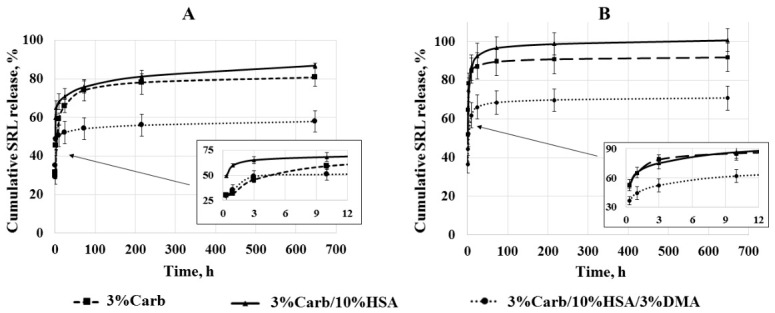
The kinetic curves of SRL release from the scaffolds. The scaffolds were incubated in PBS (**A**) and in BP (**B**) with medium replacement. The cumulative SRL release was calculated as a percentage of the SRL released from the scaffold over time compared to the initial SRL content in the scaffold. Data are presented as mean (*n* = 3) ± SD.

SRL release was studied under medium replacement conditions that mimic drug release in the bloodstream. The rate of SRL release from scaffolds in BP is higher than in PBS (Figure 3A,B). The lowest SRL release rate in BP as well as in PBS was observed for scaffold 3%Carb/10%HSA/3%DMA/SRL containing DMA. The maximal release rate was observed for 3%Carb/10%HSA/SRL. When incubated in BP, more than 80% of SRL was released from the scaffolds 3%Carb/10%HSA/SRL and 3%Carb/SRL during the first 24 h (Figure 3). During the same time, no more than 65% of the drug was released from the scaffold 3%Carb/10%HSA/3%DMA/SRL.

### 2.3. In Vitro Cytotoxicity of PU Scaffolds

**Figure 4:**
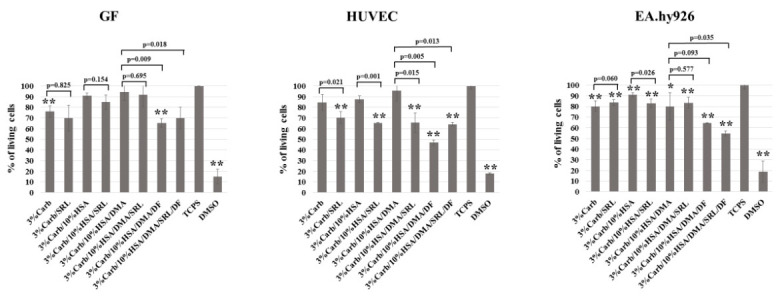
The cytotoxicity of Carb-based scaffolds in the extraction test. Pre-adhered to TCPS, GF, HUVEC, and EA.hy926 cells were cultured with extracts from scaffolds. The number of living cells cultured in fresh medium was estimated as 100%. Data are presented as mean (*n* = 3) ± SD. *, ** Statistically significant differences for a particular scaffold compared to TCPS (*—*p* < 0.05, **—*p* < 0.01). *p*-values for pairwise comparison are presented above the brackets.

The cytotoxicity of the scaffolds was tested in an extraction test according to ISO 10993-5 [17] (Figure 4). Three types of cells, GF, HUVEC, and EA.hy926, were used to study scaffolds containing 0.9 μg/cm^2^ SRL and 90 μg/cm^2^ DF. The cytotoxic effect of the drug-enriched scaffolds on cells is obviously related with the drug released from the fibers. HUVEC were more sensitive to SRL released from scaffolds to culture medium than GF and EA.hy926. The incorporation of SRL into the fibers resulted in a statistically significant decrease in the number of living cells for all types of scaffolds for HUVEC (Figure 4). All cell types were sensitive to DF. The number of viable cells decreased 1.5-fold for scaffold 3%Carb/10%HSA/DMA/DF compared to 3%Carb/10%HSA/DMA for GF and EA.hy926 cells and 2-fold—for HUVEC.

We also compared cytotoxicity of the scaffolds 3%Carb and 3%Carb/SRL with SRL concentration of 0.9 μg/cm^2^. Pre-adhered SMCs were cultured in the medium in which the scaffolds were previously incubated for 48 h. The presence of SRL in fibers did not change the percentage of living SMCs cultured in medium pre-incubated with scaffolds, which was 80.8 ± 5.8% for the tested scaffolds compared to untreated cells. It means that the concentrations of SRL released from scaffolds in 48 h were lower than cytotoxic for SMCs.

### 2.4. Interaction of Cells with Scaffolds

**Figure 5:**
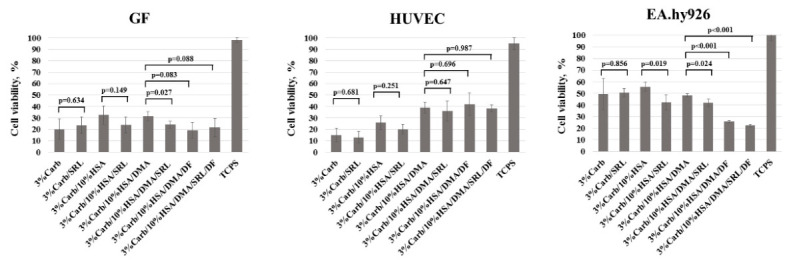
The viability of cells cultured on different scaffolds. The cells were seeded and cultured for 48 h. The number of living cells was determined using the Alamar Blue test. Cell viability on scaffolds is presented as a percentage relative to cells cultured on TCPS. Data are presented as mean (*n* = 3) ± SD. *p*-values for pairwise comparison are presented above the brackets.

Cells of different types are sensitive to the composition of the scaffolds when cells are cultivated on scaffolds. If we compare scaffolds without drugs, the viability of GF and HUVEC cells on scaffold 3% Carb was low, but it increased at least 1.5 times for scaffolds 3%Carb/10%HSA and 3%Carb/10%HSA/DMA (Figure 5). EA.hy926 cells are the less sensitive to scaffold compositions. For all the scaffolds mentioned above, the percentage of cells seeded on scaffolds varied within 45–50% compared to TCPS. GF and EA.hy926 cells are more sensitive to the drug-enriched scaffolds containing HSA and DMA than HUVEC cells (Figure 5). For most types of HSA-containing scaffolds, the viability of GF and EA.hy926 decreased when SRL and, to a greater extent, DF are incorporated into the fibers (Figure 5). A small correlation between extraction test data and viability of cells cultured on scaffolds was observed for 3%Carb, 3%Carb/SRL, 3%Carb/10%HSA, and 3%Carb/10%HSA/SRL for HUVEC (compare Figure 4 and Figure 5). No significant differences in cell viability were observed for 3%Carb/10%HSA/DMA and drug-enriched scaffolds 3%Carb/10%HSA/DMA/SRL, 3%Carb/10%HSA/DMA/DF, and 3%Carb/10%HSA/DMA/SRL/DF for HUVEC, although drug-enriched scaffolds demonstrated significantly greater cytotoxicity in the extraction test (Figure 4).

**Figure 6:**
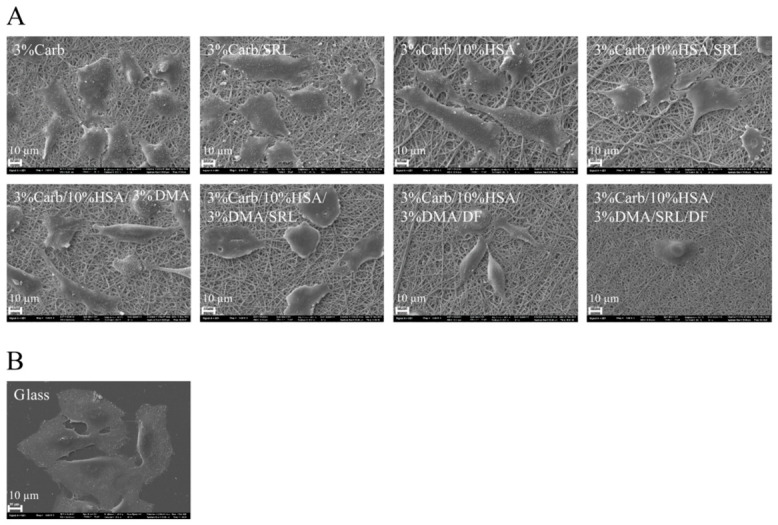
SEM images of EA.hy926 cells cultured on Carb-based scaffolds (**A**) and on glass (**B**).

Endothelial cells and SMC are the main cell types determining the long-term functionality of CVD. Since the amount of primary endothelial cell HUVEC was limited, we used the EA.hy926 cell line—somatic hybrid cells that often are used for cardiovascular disease research. To study endothelial cell adhesion, EA.hy926 cells were seeded and cultured on scaffolds with subsequent evaluation of cell morphology by SEM (Figure 6A). Cells cultured on glass were used as a control (Figure 6B). The cells were spreading on the glass, indicating adherence to the surface. The shape and size of cells cultured on scaffolds depended on the scaffold composition. Cells cultured on scaffolds 3%Carb, 3%Carb/SRL, 3%Carb/10%HSA, 3%Carb/10%HSA/SRL, and 3%Carb/10%HSA/DMA/SRL were less spread compared with glass. Cells cultured on 3%Carb/10%HSA/DMA and 3%Carb/10%HSA/DMA/DF have an elongated shape. The round cells were observed on scaffold 3%Carb/10%HSA/DMA/SRL/DF, which correlates with data on low cell viability on this scaffold (see Figure 5). The qualitative characteristics of the SEM images are presented in Table 2.

**Figure 7:**
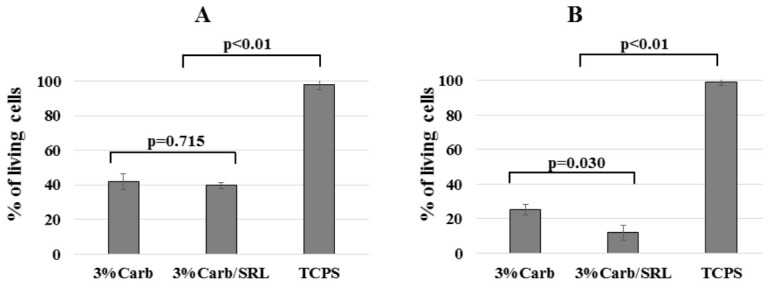
Adhesion (**A**) and proliferation (**B**) of SMCs cultured on scaffolds 3%Carb and 3%Carb/SRL. The cells were seeded on different scaffolds and cultured for 2 days for the adhesion test (**A**) or 14 days for the proliferation test (**B**). The number of living cells was determined using the Alamar Blue test. Cell viability on scaffolds is presented as a percentage relative to cells cultured on TCPS. Data are presented as mean (*n* = 3) ± SD. *p*-values for pairwise comparison are presented above the brackets.

To study adhesion and proliferation of SMCs, cells were cultured on scaffolds for 48 h and 14 days, respectively. The presence of SRL in the scaffold did not affect the adhesion of SMCs (Figure 7A). As expected, the incorporation of SRL into the fibers of PU scaffolds leads to 2-fold decrease in the SMC proliferation rate (Figure 7B). Due to the limited availability of primary SMCs and the requirement to use cells at early passages, we were unable to evaluate all SRL-enriched scaffolds in the proliferation assay.

### 2.5. Hemocompatibility

**Figure 8:**
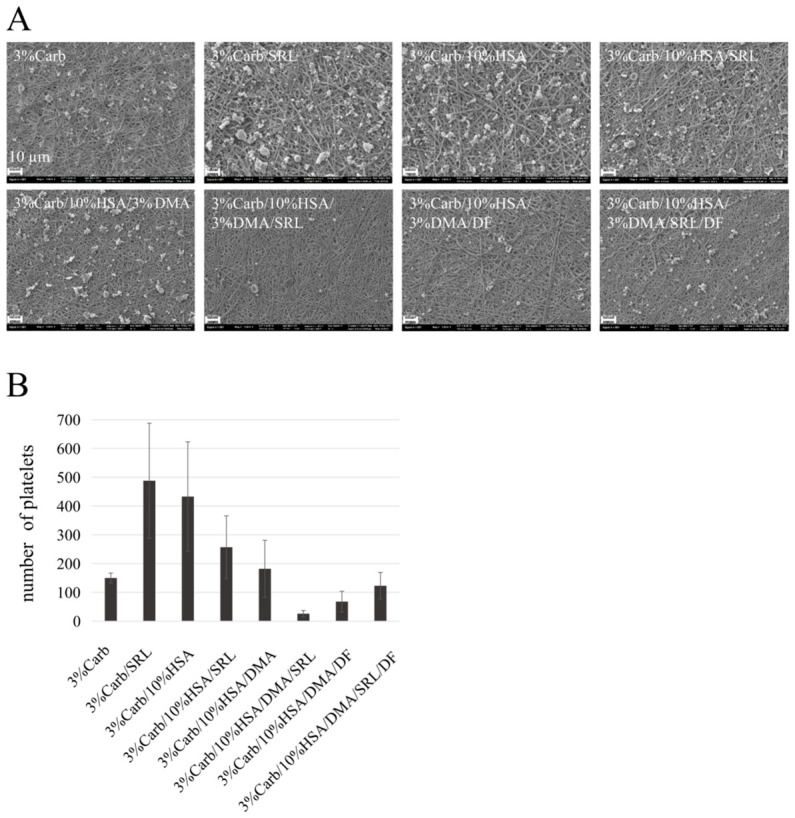
Platelet adhesion on Carb-based scaffolds. SEM images of platelets adhered on scaffolds (**A**). The number of attached platelets was assessed using SEM (**B**).

To assess the hemocompatibility of the scaffolds, the level of hemolysis and platelet adhesion were studied according to ISO 10993-4 [18]. Carb-based scaffolds were shown to not induce red blood cell destruction. The hemolytic ratio was less than 0.5% for all scaffolds. According to SEM data, maximal platelet adhesion was observed for scaffolds 3%Carb/SRL, 3%Carb/10%HSA, 3%Carb/10%HSA/SRL, and 3%Carb/10%HSA/DMA (Figure 8B). Drug-enriched scaffolds based on 3%Carb/10%HSA/DMA are less susceptible to platelet adhesion than 3%Carb/SRL and 3%Carb/10%HSA/SRL. The minimal level of platelet adhesion was observed for scaffolds 3%Carb/10%HSA/DMA/SRL and 3%Carb/10%HSA/DMA/DF. Scaffolds 3%Carb/SRL, 3%Carb/10%HSA, 3%Carb/10%HSA/SRL, and 3%Carb/10%HSA/DMA were prone to stimulate platelet aggregation (Figure 8A).

### 2.6. Cell-to-Scaffold Inflammatory Response

**Figure 9:**
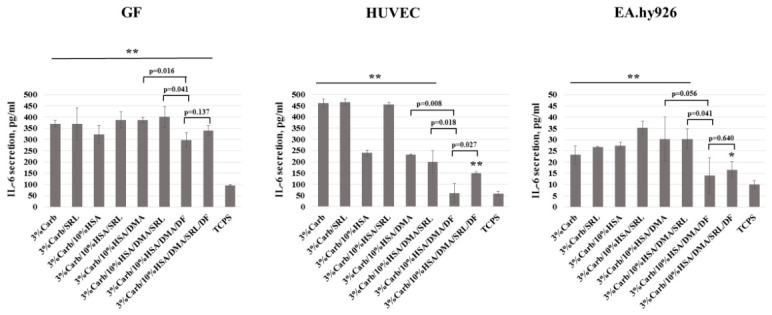
IL-6 secretion by cells. The diagram shows the concentration of IL-6 normalized to the number of cells in the well. The number of adherent cells was determined using the Alamar Blue test. Data are presented as mean (*n* = 3) ± SD. *, ** Statistically significant difference for a particular scaffold compared to TCPS (*—*p* < 0.05, **—*p* < 0.01). *p*-values for pairwise comparison are presented above the brackets.

The secretion of pro-inflammatory IL-6 by cells in response to cell-to-scaffold interaction was studied. The level of IL-6 was measured in culture medium after 48 h of cell culture on scaffolds and presented as pg/mL normalized to the number of viable cells in the well according to the Alamar Blue assay. The basic IL-6 concentration for GF, HUVEC and EA.hy926 cells cultured on TCPS was 90, 60, and 10 pg/mL, respectively (Figure 9). Cultivation on the surface of the scaffolds leads to the induction of IL-6 secretion by cells in comparison with TCPS for all cell types. In general, the level of inflammation depends on the composition of the scaffolds and the cell type. The presence of DF in scaffolds significantly reduces IL-6 secretion by GF and abrogate IL-6 secretion by HUVEC and EA.hy926 cells induced by scaffolds, returning its level close to that for cells cultured on TCPS (Figure 9).

M0 macrophages derived from THP-1 monocytes were used as a model cell to study the macrophage response to scaffolds. M1-polarized macrophages and LPS-treated macrophages were used as controls. The concentrations of pro-inflammatory cytokines Il-6 and TNF-α and anti-inflammatory Il-10 in culture media were determined by ELISA (Table 3). The concentration of TNF-α released by macrophages seeded on the 3%Carb/10%HSA/3%DMA scaffold after 24 h was 2.8 ± 0.5 pg/mL and did not change significantly after 72 h (3.5 ± 0.5 pg/mL). The concentrations of TNF-α after 24 h and 72 h for M1 macrophages were similar to those for the LPS-treated control. The TNF-α concentration in these cases increased 1.5-fold after 72 h compared with 24 h. The concentration of Il-6 for macrophages seeded on the scaffold was minimal (4.0 ± 1.2 and 5.8 ± 2.1 after 24 h and 72 h, respectively) compared to M0, M1, and LPS-treated macrophages. For M1 and LPS-treated macrophages more then 2-fold increase in IL-6 concentration after 72 h compared with 24 h was observed. The concentrations of anti-inflammatory cytokine Il-10 for M0, M1, and LPS-treated macrophages were very low (mostly below the detection limit) both after 24 h and after 72 h, while the Il-10 level for macrophages on the 3%Carb/10%HSA/3%DMA scaffold increased significantly after 72 h (from 2.5 ± 0.7 to 13.7 ± 1.2).

## 3. Discussion

Eight different Carb-based scaffolds were fabricated using ES, and their structure and mechanical properties were analyzed. It was shown that the fibers’ diameter and contact angle depend on the composition of the ES blend. The incorporation of drugs into fibers did not affect their diameter, tensile strength, or elasticity of scaffolds but increased the contact angle by 4–10%. The increased hydrophobicity (the increase in the contact angle) of drug-enriched scaffolds is likely related to the presence of hydrophobic groups in the drug structure. Tensile testing showed that all materials behave as elastomers, with the 3%Carb scaffold being the strongest. The incorporation of HSA led to a decrease in tensile strength and elongation at break, whereas DMA had no significant effect on these parameters. DMA is a high-boiling (165 °C) polar solvent [19], and its incorporation into the ES blend resulted in a decrease in fiber diameter. These data correlate with those previously obtained showing that the introduction of high-boiling DMSO in the ES blend resulted in a decrease in fiber diameter in polycaprolactone (PCL)-based scaffolds [16].

All scaffolds remained stable in PBS and in BP for 120 days. According to SEM data, no scaffold degradation or changes in fiber diameter were observed. The high elasticity under low loads and the high strength of the studied scaffolds make them suitable materials for vascular implants.

SRL release from scaffolds was studied under medium replacement conditions that mimic drug release in the bloodstream. The content of DF and SRL in scaffolds was 90 and 0.9 μg/cm^2^, respectively. These concentrations of the drugs were taken based on the toxic and active concentrations of these drugs, taking into account their release from the scaffolds [14,16,20]. In fact, the IC50 of DF for cells ranges from 200 to 600 µM [20], whereas the IC50 of SRL depends on cell type and ranges in a wide range from nM to >25 µM [21].

The rate of SRL release from scaffolds in BP was higher than that in PBS. In both PBS and BP, the rate of SRL release decreased in the following order: 3%Carb/10%HSA/SRL > 3%Carb/SRL > 3%Carb/10%HSA/3%DMA/SRL. The lowest SRL release was observed for the scaffold containing the high-boiling (165 °C) polar solvent DMA. These data correlate with previously reported data obtained for polycaprolactone (PCL)-based scaffolds [16]. It was shown that the addition of DMSO (boiling point 189 °C) to the ES blend led to the retention of SRL in the fibers. The SRL release rate decreased in the following order for the scaffolds: 5%PCL/SRL/10%HSA > 5%PCL/SRL > 5%PCL/10%HSA/3% DMSO/SRL. According to the data obtained in the present work, the scaffold 3%Carb/10%HSA/3%DMA/SRL is more suitable for long-term SRL delivery.

Unlike SRL, DF is quickly released from the studied scaffolds. Earlier, we investigated DF release kinetics from scaffolds 3%PU/10%HSA/3%DMSO/DF and 3%PU/DF [14]. Regardless of the fiber composition, up to 90% of DF was released from the scaffolds within 24 h, which we attribute to the solubility of DF. DF has a smaller molecular size and is moderately soluble in water, unlike hydrophobic SRL. Our data are consistent with the data of other authors. Wlodarczyk et al. studied the release of DF and SRL from two-component scaffolds fabricated by dual-jet ES based on poly (D,L-lactide-coglycolide) (PLGA) and polycarbonate PU (hydrophobic ChronoSil and hydrophilic HydroThane) [22]. PLGA fibers contained SRL and DF. DF was quickly released from PLGA fibers. The DF release rate from scaffolds depended on the PU used. Scaffolds consisting of pure PLGA fibers (in the absence of PU) and PLGA/HydroThane exhibited similar DF release kinetics (approximately 90% of DF was released within 72 h). DF was released from the PLGA/ChronoSil scaffold more slowly (about 60% in 72 h). The authors observed poor SRL release from the tested scaffolds (at best 33% after 84 days for PDLGA).

The structure of electrospun scaffolds mimic the extracellular matrix and provide cell adhesion in vitro [23]. Drug-free Carb-based scaffolds demonstrated minimal cytotoxic effect for all tested cell types. The cytotoxic effect of drug-enriched scaffolds on cells is evidently attributable to the drug released from the fibers. HUVEC were more sensitive to SRL released from the scaffolds to culture medium than GF and EA.hy926. All cell types were sensitive to DF in the current experimental conditions. The differential cellular responses to the drugs incorporated into the scaffolds can be explained by (1) the lower content of SRL relative to DF and/or (2) the rapid release of DF from the scaffolds. In contrast to SRL, DF is rapidly released from the fibers under the experimental conditions [10] and reaches a concentration close to cytotoxic. The estimated maximal DF concentration was approximately 750 µM. At the same time, under the experimental conditions, SRL concentration theoretically could not exceed 2 µM.

The results of viability and cytotoxicity tests demonstrate that for HUVEC, the scaffold structure and fiber composition have a greater effect on cell adhesion and viability than drugs released from the scaffolds. A weak correlation between cytotoxicity and viability was observed for scaffolds 3%Carb, 3%Carb/SRL, 3%Carb/10%HSA, and 3%Carb/10%HSA/SRL. No significant differences in HUVEC viability were observed for 3%Carb/10%HSA/DMA and the drug-enriched scaffolds 3%Carb/10%HSA/DMA/SRL, 3%Carb/10%HSA/DMA/DF, and 3%Carb/10%HSA/DMA/SRL/DF. However, the drug-enriched scaffolds exhibited significantly higher cytotoxicity. These data correlate with our previous data [10]. Although the 3%Carb/10%HSA/3%DMSO/DF scaffold had a moderate cytotoxic effect on HUVEC compared to the 3%Carb/10%HSA/3%DMSO scaffold, the viability of HUVEC cultured on these scaffolds did not differ significantly. Viability depends not only on cell death caused by cytotoxic components released from the material but also on cell adhesion, proliferation, and metabolism. When a potentially toxic component is gradually released from the material, it can be metabolized in cells and its real concentration in culture medium and in cells decreases without reaching cytotoxic values.

GF and EA.hy926 cells cultured on PU scaffolds were more sensitive to the drug-enriched scaffolds containing HSA and DMA than HUVEC. For most types of HSA-containing scaffolds, the viability of GF and EA.hy926 decreased when SRL and, to a greater extent, DF were incorporated into the fibers. These data are consistent with our previous study of DF-enriched Carb-based scaffolds [14]. The viability of GF on the scaffold 3%Carb/10%HSA/3%DMSO/DF containing DF at a concentration of 90 μg/cm^2^ was 1.5-fold lower than the viability of the scaffold 3%Carb/10%HSA/3%DMSO without DF. The viability of HUVEC, on the contrary, did not depend on the presence of DF in fibers, although the extraction test showed a negative effect of DF on HUVEC cells cultured in medium pre-incubated with DF-enriched scaffolds. Earlier, Wlodarczyk and colleagues showed that the incorporation of SRL in dual-jet electrospun poly(D,L-lactide-co-glycolide)/polycarbonate PU scaffolds affected the viability and adhesion of human fibroblasts WI-38 on PDLGA/PU scaffolds [22]. The decrease in viability and adhesion correlated with the rate of SRL release from the scaffolds and was due to cytotoxicity caused by SRL. Notably, the SRL concentration in PDLGA/PU scaffolds was two orders of magnitude higher than that used in our Carb-based scaffolds.

We have shown that the presence of SRL at a dose of 0.9 μg/cm^2^ in the fibers did not affect the adhesion of smooth muscle cells but reduces SMC proliferation. The concentration of SRL released from scaffolds was lower than cytotoxic concentrations for SMC but sufficient to inhibit their proliferation. As expected, the incorporation of SRL into the fibers of PU scaffolds led to a 2-fold decrease in SMC proliferation rate.

The materials intended for vascular implants should be effectively endothelialized. Endothelialization of blood-contacting surfaces prevents platelet adhesion and recruitment leukocytes to the stented area [24] and also decreases the risk of stent thrombosis [25,26]. The endothelium plays an important role in the proliferation of vascular smooth muscle cells, the inflammatory process, and thrombus formation in the stent area [27]. The scaffolds 3%Carb, 3%Carb/SRL, 3%Carb/10%HSA, 3%Carb/10%HSA/SRL, and 3%Carb/10%HSA/DMA/SRL are effectively endothelialized. The shape and size of EA.hy926 cells cultured on them are comparable with those cultured on glass. Cells cultured on DF-containing scaffolds exhibited an elongated or round shape. The cell viability on these scaffolds was also minimal.

Biomaterials can induce thrombosis via protein adsorption, platelet adhesion and activation, the stimulation of coagulation, and leukocyte and complement activation [28]. Therefore, we investigated the effect of PU-based scaffold composition on platelet adhesion and hemolysis. According to the literature, HSA increases the hemocompatibility of the materials [29,30]. This property is especially important for materials in contact with blood. The hemolysis ratio was less than 0.5% for all scaffolds. This level of hemolysis meets the requirements of the ISO 7198 standard [31]. The incubation of platelet-rich plasma (PRP) on scaffolds 3%Carb/SRL, 3%Carb/10%HSA, 3%Carb/10%HSA/SRL, and 3%Carb/10%HSA/DMA resulted in high platelet adhesion and aggregation. The minimal level of platelet adhesion was observed for scaffolds 3%Carb/10%HSA/DMA/SRL and 3%Carb/10%HSA/DMA/DF. These results demonstrated that Carb-based scaffolds are hemocompatible and could be used for the fabrication of vascular implants.

One of the problems of using synthetic materials in vascular surgery is the induction of a cellular immune response. Recent studies have demonstrated that the immune/inflammatory response induced by tissue-engineered vascular grafts might involve platelet activation and inflammation immediately after implantation [32]. The type and duration of the inflammatory response can affect tissue regeneration, and chronic inflammation can cause restenosis. Therefore, it is important to consider how the properties of the material affect the inflammatory response in cells. We studied the response of endothelial cells (EC) and fibroblasts cultured on PU scaffolds as well as macrophages obtained from THP-1 monocytes. EC participate in both innate and adaptive immune responses [33]. EC possess innate immune functions, including cytokine secretion. GF cells are considered “nonclassical” innate immune cells. GF can produce inflammatory cytokines in response to bacteria and damage-related signals [34]. The inflammatory response of both these cell types was assessed by the secretion of IL-6—one of the most active cytokines involved in the immune response and inflammation [35].

Cultivation of all cells on the surface of the scaffolds leads to the induction of IL-6 secretion by cells in comparison with TCPS. In general, the level of inflammation depends on the composition of the scaffolds and the cell type. The IL-6 level showed that the Carb-based scaffolds stimulate an inflammatory response of fibroblasts and endothelial cells cultured on these scaffolds. The initial induction of pro-inflammatory cytokines may be beneficial for recruiting other cells and promoting the release of cytokines that can stimulate regenerative processes in vivo [36]. On the other hand, recent studies have demonstrated that inflammation increases the risk of postoperative complications and is one of the main causes of restenosis after stenting [32,37]. Scaffolds loaded with diclofenac, a nonsteroidal anti-inflammatory drug, may serve to reduce inflammation [38]. It was shown that the introduction of DF into scaffolds suppresses the induction of inflammation. For cells cultured on scaffolds 3%Carb/10%HSA/DMA/DF and 3%Carb/10%HSA/DMA/SRL/DF, the IL-6 concentration is close to that for cells cultured on TCPS. However, scaffolds containing DF are more cytotoxic to the tested cells.

We evaluated the in vitro immune response induced by the 3%Carb/10%HSA/DMA scaffold. This scaffold was selected to test the effect on macrophage polarization since IL-6 secretion by cells (GF, HUVEC and EA.hy926) cultured on this scaffold increased compared to that for TCPS. We compared the concentrations of pro-inflammatory cytokines IL-6 and TNF-α and anti-inflammatory IL-10 in cell-free culture media for M0 macrophages cultured on the scaffold, M1-polarized macrophages, and LPS-treated macrophages. M0 macrophages were derived from THP-1 monocytes, which are widely used as a model to study the immune response of monocyte-derived macrophages [39]. A significant increase in the concentration of anti-inflammatory IL-10 and small changes in the concentrations of pro-inflammatory IL-6 and TNF-α for macrophages seeded on scaffold compared with M1 and LPS-treated macrophages suggest that the 3%Carb/10%HSA/3%DMA scaffold promotes M2 anti-inflammatory macrophage polarization rather than an M1 pro-inflammatory response.

The data obtained demonstrate that Carb-based scaffolds could be used as a material for CVS that promotes an anti-inflammatory response. Nevertheless, additional detailed studies of the effect of PU-scaffolds on macrophage modulation are required. Earlier, Pisani and colleagues hypothesized that ES-fabricated scaffolds can induce a pro-regenerative response capable of supporting and improving tissue replacement, preventing chronic inflammation [36]. The authors suggest that (1) the induction of pro-inflammatory cytokine release during the first 24 h could be useful for recruiting other cells and stimulating the regenerative process and that (2) an anti-inflammatory response within 3 days promotes further tissue replacement. If this suggestion is correct, then the 3%Carb/10%HSA/DMA scaffold is a promising material for CVS. If the inflammatory response of macrophages within the first 24 h is undesirable, then DF-enriched scaffolds could be used. We previously demonstrated that DF is almost completely released from the scaffolds 3%Carb/10%HSA/3%DMSO/DF and 3%Carb/DF within 24 h and could suppress acute inflammation after implantation [14].

Several limitations of the present study should be acknowledged. First, all experiments were conducted in vitro using human primary cells and cell lines. While these models provide valuable insights, they do not recapitulate the complex biological environment in vivo. Therefore, animal implantation studies are necessary to evaluate an SRL-enriched stent covering efficacy. Second, the long-term biological stability of the Carb-based scaffolds, including their degradation profile and potential loss of mechanical integrity over time, is needed to be assessed in vivo. A comprehensive in vivo study into the function of the stents covered with Carb-based scaffolds in a prolonged follow-up is necessary in order to assess the efficiency of the scaffolds. The most promising scaffold, 3%Carb/10%HSA/DMA/SRL, should be compared to the clinically used stents. Future studies should focus on validating the anti-proliferative and anti-inflammatory effects in vivo and assessing scaffold stability under physiological conditions for extended periods.

## 4. Materials and Methods

### 4.1. Fabrication of Scaffolds by Electrospinning

The ES blends were prepared using solutions of Carbothane™ AC-4075A (Lubrizol, Wilmington, MA, USA) and human serum albumin (Sigma-Aldrich, St. Louis, MO, USA) in 1,1,1,3,3,3-hexafluoroisopropanol (Sigma-Aldrich, St. Louis, MO, USA). The HSA concentration in the scaffolds was 10% (*w*/*w*). Diclofenac (Sigma-Aldrich, St. Louis, MO, USA) and sirolimus (150,301, Fujian Kerui Pharmaceutical Co. Ltd., Fujian, China) were dissolved in 1,1,1,3,3,3-hexafluoroisopropanol and added to the ES blend to achieve a final concentration in the scaffolds of 90 and 0.9 μg/cm^2^, respectively. Dimethylacetamide (Sigma-Aldrich, St. Louis, MO, USA) was added to the solution of polymers to the final concentration of 3% (*v*/*v*).

Scaffolds of 60–100 µm thickness were fabricated using an electrospinning device (model NF-103, MECC Co., Ltd., Fukuoka, Japan) under the following conditions: voltage 16–18 kV and feed rate 1.2–1.4 mL/h (these parameters were optimized for each scaffold composition); capillary–collector distance—15 cm; needle—22G; collector rotation speed—300 rpm; temperature—25 °C; and humidity—25–30%. After fabrication, the scaffolds were removed from the collector, dried, and stored in sealed ziplock polyethylene containers at 4 °C.

### 4.2. Evaluation of Scaffolds Structure and Physicochemical Properties

The scaffold structure was analyzed via scanning electron microscopy (SEM). The scaffolds were fixed on a sample holder using double-sided carbon tape before Au/Pd sputter-coating and analyzed using an EVO 10 scanning electron microscope (Carl Zeiss AG, Oberkochen, Germany) at an accelerating voltage of 10 kV. The fiber diameter was assessed from SEM images.

Strain–stress diagrams were obtained using a universal Zwick/Roell Z100 (Zwick Roell, Ulm, Germany) test bench. Four 1 × 5 cm rectangular sheets for each matrix were cut and placed between holders at a distance of 2–2.5 cm. The tensile strength testing was performed at a rate of 10 mm × min^−1^ at room temperature (21–23 °C). Tensile strength was calculated by the ratio (maximum applied force)/(initial cross section); elongation at break was calculated as ((length at break − initial length)/initial length) × 100.

To analyze the stability of the scaffold structure in different mediums, the scaffolds were cut into disks with a diameter of 10 mm and incubated in 400 µL of PBS or EDTA-stabilized human blood plasma (BP) at 37 °C for 30 and 120 days in the presence of 100 μg/mL penicillin and streptomycin (Invitrogen, Grand Island, NY, USA). The medium (PBS or BP) was replaced once a month. This work was approved by the Local Ethical Committee of the Center of Personalized Medicine ICBFM SB RAS (No 8, 7 July 2020). After incubation, the specimens were washed twice with water and analyzed via SEM.

The contact angle was measured on a Drop Shape Analyzer–DS A25 (Kruss GmbH, Hamburg, Germany) using water as a solvent (drop volume—1 μL; shooting speed—160 frames per second).

### 4.3. SRL Release

^3^H-SRL was synthesized by thermoactivated tritium exchange and purified, as described earlier [16]. ^3^H-SRL was mixed with unlabeled SRL to achieve a final concentration of SRL in the scaffolds of 90 and 0.9 μg/cm^2^ with radioactivity of 20,000–25,000 cpm/cm^2^. The scaffolds were fabricated as described above.

To evaluate SRL release, disks with a diameter of 10 mm (~0.785 cm^2^) were cut out of the scaffolds by die cutting and placed in 1.5 mL tubes. Then, 400 µL of PBS or EDTA-stabilized BP was added to each tube. All samples were studied in duplicate. The tubes were incubated on a TS-100 thermoshaker (Vector-Best, Novosibirsk, Russia) with a platform rotation speed of 200 rpm at 37 °C. SRL release was studied with medium replacement: the supernatant was removed at each time point (20 min, 60 min, 3 h, 9 h, 27 h, 3 days, 9 days, and 27 days), fresh solution was added, and the scaffold was incubated in the fresh medium until the next time point. An amount of 100 µL of the supernatant was thoroughly mixed with 900 µL “ULTIMA GOLD LTT” scintillator (Perkin Elmer, Waltham, MA, USA), and radioactivity of each sample was measured in duplicate on a Tri-Carb 2800 TR-counter (PerkinElmer, Waltham, MA, USA).

### 4.4. Cells Used in the Current Study

To study in vitro cytotoxicity by extraction test EA.hy926 cells (ATCC, Manassas, VA, USA), primary human umbilical vein endothelial cells (HUVECs), obtained as described in [40], and gingival fibroblasts (GF), obtained from human gingiva as described in [14], were used. The study was approved by the Local Ethical Committee of the Center of Personalized Medicine ICBFM SB RAS (No 8, 7 July 2020). To obtain primary cells, informed consent was obtained from donors before inclusion in the study. Primary cells were used in experiments at passages 2–5. All cells mentioned above were cultured in IMDM (Invitrogen, Carlsbad, CA, USA) supplemented with 10% fetal bovine serum (FBS) and 100 μg/mL penicillin and streptomycin in CO_2_ in incubator at 37 °C and 5% CO_2_ and reseeded upon reaching 70–80% monolayer.

Vascular smooth muscle cells (SMCs) were used to test the effect of drug-enriched scaffolds on cell adhesion and proliferation. SMCs were derived from a fragment of the internal carotid artery removed during surgical correction of arterial kinking. The tissue specimens were washed with PBS, finely minced, and incubated in collagenase II. They were then transferred to Petri dishes and, after three days, the samples were washed three times with IMDM to eliminate tissue debris. Fresh IMDM supplemented with FBS was then added, and the cells were incubated at 37 °C in a 5% CO_2_ atmosphere. The culture medium was replaced every five days over a period of 20–25 days, until the cells reached approximately 80% confluence. The smooth muscle cells (SMC) were maintained in IMDM containing FBS and were passaged using 0.2% collagenase II solution. The procedure was accompanied by obtaining informed consent and was approved by the Ethical Committee. Cells were cultured and reseeded as described above.

To monitor the effect of the scaffold on macrophage activation, human monocytic leukemia cells THP-1 (American Type Culture Collection, Manassas, VA, USA) were used to obtain macrophages M0. THP-1, cells were cultured in a 25-flask in RPMI-1640 culture medium (Gibco, Paisley, UK) supplemented with 10% FBS and 100 μg/mL penicillin and streptomycin (RPMI complete medium) at 37 °C and 5% CO_2_, refreshing medium every 48 h. The cell density did not exceed 10^6^ cells per 1 mL. The cells were cultured until the required amount was obtained for the experiment.

### 4.5. In Vitro Cytotoxicity, Extraction Test

To obtain the extracts, discs with a diameter of 10 mm were incubated with 300 µL of complete IMDM medium at 37 °C and 5% CO_2_ for 24 h according to ISO 10993-12 [41]. GF, EA.hy926, and HUVEC cells were seeded into a 96-well plate with 1500, 4000, and 4000 cells per well, respectively, and cultured in complete IMDM medium in a CO_2_ incubator at 37 °C and 5% CO_2_ for 24 h to allow the cells to attach. After 24 h, after cell adhesion, the medium in the wells was replaced with 100 µL of medium in which the scaffolds were incubated. As a negative control, cells were cultured in fresh complete medium. As a positive control of cell death, cells were cultured in complete medium supplemented with 5% DMSO. The number of living cells was determined after 24 h using the Alamar Blue assay.

### 4.6. Interaction of Cells with Scaffolds

Disks with a diameter of 10 mm were excised from the scaffolds by die cutting and fixed to the bottom of the wells of 48-well plates using Teflon rings. The disks were pre-incubated with 50 µL of IMDM medium for 30 min to achieve complete moistening. GF, EA.hy926, and HUVEC cells were seeded on scaffolds in an amount of 3000, 8000, and 8000 cells/well, respectively, in 300 µL of growth medium and cultured for 48 h at 37 °C and 5% CO_2_.

SMCs were seeded on scaffolds in an amount of 4000 cells/well and cultured in 300 µL of complete medium for 48 h at 37 °C and 5% CO_2_ for the adhesion test. For the proliferation test, SMCs were cultured for 14 days by adding 100 µL of fresh medium every three days.

Cells cultured on the surface of 48-well polystyrene culture plates (TCPS) without scaffolds were used as a control. To obtain a calibration curve, different numbers of cells were seeded on TCPS. The number of living cells was determined using the Alamar Blue assay (Invitrogen, Frederick, MD, USA) in accordance with the manufacturer’s recommendations.

To study the effect of scaffolds on the morphology of EA.hy926, the cells were cultured as described above for 48 h. The cells cultured on round cover glasses (Menzel-Glaser, Thermo Fisher Scientific, Braunschweig, Germany) were used as control. Then, culture medium was removed, and scaffolds were washed twice with 500 µL of PBS to remove non-adherent cells and were fixed overnight with 2% buffered glutaraldehyde. Fixed samples were washed with 500 µL of PBS 3 times for 5 min with subsequent serial dehydration in 500 µL of 50% ethanol (2 × 10 min incubation), then in 500 µL of 70% ethanol (2 × 10 min), in 500 µL of 95% ethanol (2 × 10 min), in 500 µL of 100% ethanol (3 × 15 min), in 300 µL of 2:1 mixture of 100% ethanol and hexamethyldisilazane (HMDS, Sigma-Aldrich, Steinheim, Germany) (15 min), in 300 µL of 1:1 mixture of 100% ethanol and HMDS (15 min), in 300 µL of 1:2 mixture of 100% ethanol and HMDS (15 min), and in 300 µL of 100% HMDS (3 × 15 min). Then, scaffolds were dried in air at room temperature, sputter coated with gold/palladium, and analyzed via SEM. ImageJ (1.54g version) software was used to quantitatively analyze the cell spreading area, number of cells in the field of view, and cell coverage rate.

### 4.7. Hemocompatibility Study

Blood from healthy donors was taken in a BD vacutainer containing 0.129 M sodium citrate (BD Diagnostics Inc., Franklin Lakes, NJ, USA). This study was approved by the Local Ethical Committee, and informed consent was obtained from donors before inclusion in the study.

#### 4.7.1. Hemolysis Assay

Citrated blood was diluted with PBS in a ratio of 4:5. Discs with a diameter of 10 mm were pre-incubated in 5 mL PBS for 3 h at 25 °C in tubes. Next, 0.1 mL of diluted blood was added to each tube containing either 5 mL of PBS with pre-incubated disks, 5 mL of distilled water (positive control), or 5 mL of PBS (negative control) and incubated for 1 h at 37 °C. Then, all samples were centrifuged at 1000 rpm for 10 min. The absorbance at 540 nm was measured using a Genesys 10uv spectrophotometer (Thermo Electron Corporation, Madison, WI, USA). The hemolytic ratio was calculated as [(ODsample − ODneg)/(ODpos − ODneg)] × 100%. Each scaffold was tested in duplicate.

#### 4.7.2. Platelet Adhesion Test

Fresh blood from healthy donors was centrifuged at 200× *g* for 20 min to obtain platelet-rich plasma (PRP). The scaffold disks with a diameter of 10 mm were placed in a 48-well plate and pre-incubated with 100 µL of PBS for 30 min to achieve complete moistening, and then, PBS was removed. Next, disks were incubated with 300 μL PRP for 1 h at 37 °C. The scaffolds were washed twice with 500 µL of PBS to remove non-adherent platelets and were fixed overnight with 2% buffered glutaraldehyde. Then, samples were washed and treated with HMDS as described above for EA.hy926 cells. Then, scaffolds were dried, coated with gold/palladium, and analyzed via SEM. The number of adherent platelets was estimated from SEM images.

### 4.8. Study of Cell Inflammatory Reactions Induced by Scaffolds

GF, EA.hy926, and HUVEC cells were cultured on scaffolds for 48 h as descried above; then, the medium was collected. Control cells were cultured in the wells of 48-well plates without scaffolds.

The concentration of IL-6 in the culture medium was determined in duplicates of 100 µL each by enzyme-linked immunosorbent assay (ELISA) using the commercial IL-6-ELISA-BEST kit (Vector-Best, Novosibirsk, Russia) in accordance with the manufacturer’s recommendations. The concentration of IL-6 was normalized to the number of cells in the each well. The number of adherent cells was determined using the Alamar Blue assay.

THP-1 cells (1.5 × 10^5^ cell per well) were seeded directly on 3%Carb/10%HSA/3%DMA scaffold disks (d = 10 mm) in 350 μL RPMI complete medium with 50 ng/mL Phorbol 12-myristate 13-acetate (PMA, Sigma-Aldrich, Steinheim, Germany) in 48-well plates and cultured at 37 °C and 5% CO_2_ for 48 h for M0 differentiation. The medium was then replaced with a fresh complete medium without PMA and incubated for 24 h. Then, medium was replaced with 500 μL of fresh RPMI complete medium, and concentrations of Il-6, TNF-α, and Il-10 were determined after 24 h and 72 h incubation using the commercial kits IL-6-ELISA-BEST, TNF-α-ELISA-BEST and IL-10-ELISA-BEST (Vector-Best, Novosibirsk, Russia) in accordance with the manufacturer’s recommendations. The concentrations of cytokines were normalized to the number of cells.

For M0 control, THP-1 cells (4.5 × 10^5^ cells/mL) were incubated in RPMI complete medium with 50 ng/mL PMA in the wells of 48-well plates without scaffolds. The medium was then replaced with a fresh complete medium without PMA and incubated for 24 h to achieve adherent M0 macrophages. For the LPS positive control, cells were seeded in 48-well plates and treated with 1 μg/mL lipopolysaccharide (LPS, Sigma-Aldrich, St. Louis, MO, USA) after M0 differentiation. M0 macrophages were treated with 20 ng/mL recombinant human interferon gamma (IFN-γ, PeproTech, Rocky Hill, NJ, USA) and 10 pg/mL LPS to obtain M1-polarized macrophages. Supernatants, obtained from M0, M1, and LPS-treated macrophages, were used as control.

### 4.9. Statistical Analysis

The data processing was performed using the Statistica (version 7.0) package (StatSoft, Inc., Tulsa, OK, USA) and the Microsoft Excel functions. Data were calculated as mean (*n* = 3) ± standard deviation (SD). *p*-values for pairwise comparison were estimated using Student’s *t*-test between groups.

## 5. Conclusions

PU-based scaffolds were fabricated via electrospinning from blends of Carbothane™ AC-4075A with HSA, DMA, and the drugs SRL and DF. The scaffolds demonstrated favorable mechanical properties and are therefore expected to be suitable materials for vascular implants. According to SEM data, all scaffolds remained stable in PBS and BP for 120 days.

All scaffolds were hemocompatible and did not cause hemolysis. Biocompatibility and the inflammatory response induced by scaffolds were investigated on human gingival fibroblasts, human umbilical vein endothelial cells, and the EA.hy926 endothelial cell line. The viability of cells cultured on Carb-based scaffolds depends on both scaffold composition and cell type. The cultivation of endothelial cells and fibroblasts on the scaffold surface induced the secretion of pro-inflammatory interleukin IL-6 by cells. The level of inflammation depended on the cell type and scaffold composition. The incorporation of DF into the scaffolds decreased the concentration of IL-6 in the culture medium.

The obtained data demonstrate that the Carb-based scaffolds are biocompatible and possess favorable mechanical properties; therefore, they are expected to be suitable material for vascular implants. The scaffold 3%Carb/10%HSA/DMA/SRL is the most promising candidate for stent coating and vascular graft fabrication, as it is non-cytotoxic, supports endothelization, exhibits favorable mechanical properties, shows low platelet adhesion, and is associated with a relatively low level of pro-inflammatory IL-6 secretion in vitro.

## Figures and Tables

**Table 1:** Physical properties of the scaffolds.

Scaffold Type (Composition)	Thickness (µm)	Tensile Strength (MPa)	Elongation at Break (%)	Fiber Diameter (nm)			Contact Angle (°)
				Initial Scaffold	Scaffold Incubated (30 Days/120 Days) with		
					PBS	BP	
3%Carb	62 ± 9	24.6 ± 1.7	427 ± 14	666 ± 213	600 ± 174/ 607 ± 149	689 ± 214/ 620 ± 160	112.87 ± 1.66
3%Carb/SRL	69 ± 9	*	**	683 ± 166	665 ± 155/ 676 ± 177	639 ± 178/ 633 ± 185	124.28 ± 1.32
3%Carb/10%HSA	71 ± 8	15.5 ± 2.5	333 ± 44	635 ± 189	578 ± 218/ 651 ± 193	573 ± 232/ 621 ± 220	109.64 ± 1.85
3%Carb/10%HSA/SRL	72 ± 8	*	**	613 ± 168	656 ± 230/ 695 ± 240	611 ± 209/ 598 ± 165	120.81 ± 2.37
3%Carb/10%HSA/3%DMA	80 ± 10	15.0 ± 1.0	355 ± 34	508 ± 125	596 ± 179/ 610 ± 176	624 ± 187/ 600 ± 196	117.00 ± 0.97
3%Carb/10%HSA/3%DMA/SRL	75 ± 10	*	**	559 ± 120	568 ± 172/ 620 ± 172	613 ± 166/ 634 ± 183	122.89 ± 2.28
3%Carb/10%HSA/3%DMA/DF	85 ± 17	*	**	566 ± 135	539 ± 117/ 535 ± 132	653 ± 141/ 588 ± 181	123.87 ± 1.13
3%Carb/10%HSA/3%DMA/SRL/DF	95 ± 16	*	**	511 ± 110	579 ± 133/ 553 ± 141	590 ± 138/ 554 ± 129	120.75 ± 2.17

*, **—incorporation of SRL and DF does not affect the change in tensile strength and elongation at break, as it was found in pilot experiments, and therefore, these parameters were not determined in the working experimental set.

**Table 2:** The qualitative characteristics of the SEM images.

Scaffold Type	Cell Spreading Area (nm)	Number of Cells	Cell Coverage Rate (%)
3%Carb	504.3 ± 338.5	88 ± 11	17.3 ± 0.4
3%Carb/SRL	704.2 ± 382.3	92 ± 9	25.1 ± 0.7
3%Carb/10%HSA	675.2 ± 329.9	89 ± 8	23.3 ± 0.5
3%Carb/10%HSA/SRL	688.8 ± 355.2	56 ± 10	13.4 ± 0.6
3%Carb/10%HSA/3%DMA	755.6 ± 358.3	59 ± 6	16.3 ± 0.4
3%Carb/10%HSA/3%DMA/SRL	737.9 ± 458	48 ± 9	12 ± 0.9
3%Carb/10%HSA/3%DMA/DF	658.9 ± 429.3	17 ± 4	2.9 ± 0.4
3%Carb/10%HSA/3%DMA/SRL/DF	667.2 ± 456.3	2 ± 1	0.3 ± 0.2
glass	1725.3 ± 1041.6	42 ± 10	25.9 ± 1.0

**Table 3:** The concentration of cytokines TNF-α, Il-6, and IL-10 secreted by macrophages after 24 h and 72 h determined by ELISA. Results were achieved for unpolarized macrophages (M0), M1-polarized macrophages, LPS-treated cells (positive control), and macrophages cultured on the 3%Carb/10%HSA/3%DMA scaffold. Data are presented as mean (*n* = 3) ± SD. ND—not detected (values below the assay detection limit).

	TNF-α (pg/mL)		Il-6 (pg/mL)		IL-10 (pg/mL)	
	24 h	72 h	24 h	72 h	24 h	72 h
M0 (TCPS)	2.8 ± 0.3	3.1 ± 0.6	20.7 ± 3.5	28.2 ± 4.7	ND	ND
M1 (TCPS)	4.2 ± 0.5	6.4 ± 0.4	14.0 ± 2.4	37.3 ± 6.8	ND	2.6 ± 1.4
LPS-treated control (TCPS)	4.2 ± 0.3	6.1 ± 0.3	24.0 ± 3.2	57.4 ± 7.0	ND	ND
3%Carb/10%HSA/3%DMA	2.8 ± 0.5	3.5 ± 0.5	4.0 ± 1.2	5.8 ± 2.1	2.5 ± 0.7	13.7 ± 1.2

## Data Availability

The data supporting the findings of this study are included in the article. Further inquiries can be directed to the corresponding author.

## Abbreviations

The following abbreviations are used in this manuscript:

PU	Polyurethane
ES	Electrospinning
CVD	Cardiovascular devices
Carb	Carbothane
HSA	Human serum albumin
DMA	Dimethylacetamide
SRL	Sirolimus
DF	Diclofenac
BP	Blood plasma
HUVEC	Human umbilical vein endothelial cells
CVS	Cardiovascular surgery
PCL	Polycaprolactone
NSAID	Nonsteroidal anti-inflammatory drugs
HFIP	1,1,1,3,3,3-hexafluoroisopropanol
SEM	Scanning electron microscopy
FBS	Fetal bovine serum
SMC	Smooth muscle cell
TCPS	Tissue culture polystyrene
HMDS	Hexamethyldisilazane
PRP	Platelet-reach plasma PRP
ELISA	Enzyme-linked immunosorbent assay
LPS	Lipopolysaccharide
